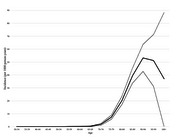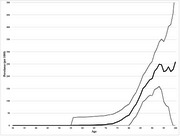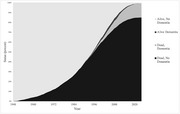# The Incidence and Prevalence of Dementia over the Life Course of Men ‐ The Manitoba Follow Up Study

**DOI:** 10.1002/alz70860_097001

**Published:** 2025-12-23

**Authors:** Philip D St. John, Carly Scramstad, Robert Tate

**Affiliations:** ^1^ University of Manitoba, Winnipeg, MB, Canada

## Abstract

**Background:**

There are few long term cohort studies over the life course of a closed population. The cumulative lifetime risk of dementia is therefore unknown. The aims of these analyses are to report: 1. The age specific incidence of dementia in a cohort study of aging men; 2. The age specific prevalence of dementia; and 3. The life course cumulative risk of dementia considering the competing risk of mortality.

**Method:**

The Manitoba Follow Up Study is a prospective cohort study of men who qualified for air crew training in the Royal Canadian Air Force during the Second World War who have been followed to the present day. The cohort was sealed in July 1948, with a mean age of 31 years old. We analyze the risk of dementia to July 2024. Dementia was determined by diagnostic reports from physicians, hospital records and long term care records, and death reviews. We calculated the incidence of dementia and 95% confidence interval (CI) in five year age groups, and calculated the point prevalence and 95% CI of dementia from age 30 to age 100. We graphically present the status of the cohort from 1948 to 2024 to visualize the risk of dementia and the competing risk of death.

**Results:**

Of the 3983 participants, 3960 died, 7 were lost to follow up, and 570 were diagnosed with dementia. The incidence of dementia was low prior to the age of 80, increasing to 20 cases per 1000 person‐years at age 80‐84 and 53 cases per 1000 person‐years at age 90‐94 (Figure 1). The point prevalence of dementia was also very closely related to age (Figure 2). Figure 3 shows the risk of dementia in the face of the competing risk of death.

**Conclusion:**

The risk of dementia is highly concentrated in older adults, and there is a high competing risk of death.